# The role of microRNAs in cardiovascular disease associated with the consumption of ultra-processed foods: a comprehensive review

**DOI:** 10.3389/fnut.2026.1790304

**Published:** 2026-04-20

**Authors:** Shengzhou Wen, Dushyantha T. Jayaweera, George R. Marzouka, Chunming Dong

**Affiliations:** 1 Department of Medicine, University of Miami Miller School of Medicine, Miami, FL, United States; 2 Interdisciplinary Stem Cell Institute, University of Miami Miller School of Medicine, Miami, FL, United States; 3 Schiff Center for Liver Diseases, University of Miami Miller School of Medicine, Miami, FL, United States; 4 Section of Infectious Diseases, Department of Medicine, University of Miami Miller School of Medicine, Miami, FL, United States; 5 Section of Cardiology, Department of Medicine, Miami VA Health System, Miami, FL, United States

**Keywords:** atherosclerosis, cardiovascular disease, extracellular vesicles, inflammation, microRNA, nutrigenomics, ultra-processed foods

## Abstract

Ultra-processed foods (UPFs) now dominate dietary intake in many countries and are consistently associated with higher risks of cardiovascular disease (CVD), including myocardial infarction, stroke, and heart failure. Beyond excess sodium, sugar, and unhealthy fats, UPFs may exert cardiovascular harm through food matrix disruption, processing-generated toxicants, additive exposure, and microbiome perturbation. These upstream insults converge on inflammatory, oxidative, and metabolic signaling pathways that regulate microRNAs (miRNAs), a class of small non-coding RNAs that orchestrate post-transcriptional gene expression across endothelial cells, vascular smooth muscle cells, macrophages, platelets, and metabolic tissues. In this review, we propose a unifying mechanistic framework in which UPF exposure reshapes both intracellular and extracellular vesicle (EV)-associated miRNA networks, thereby linking gut, liver, adipose tissue, and the vascular wall in a feed-forward cardiometabolic signaling loop. We synthesize evidence across epidemiology, experimental models, and human dietary intervention studies, while explicitly distinguishing established, emerging, and speculative mechanisms to avoid over-interpretation. We further discuss translational opportunities, including circulating miRNA/EV-miRNA biomarkers, nutritionally responsive miRNA signatures, and miRNA-targeted therapeutics. Together, this framework positions the UPF–miRNA/EV axis as a plausible molecular bridge between modern dietary exposure and atherosclerotic disease progression, and highlights priority areas for mechanistic validation and clinical translation.

## 1 Introduction

Cardiovascular disease (CVD) remains the leading cause of mortality worldwide, and diet is one of the most important modifiable determinants of long-term cardiovascular risk. Among contemporary dietary exposures, ultra-processed foods (UPFs) warrant particular attention because they now contribute a substantial share of caloric intake in many populations and have been consistently associated with incident CVD independent of total energy intake or macronutrient composition (1–8). Beyond excess sodium, sugar, and unhealthy fats, UPFs may also promote cardiovascular injury by disrupting the food matrix, increasing exposure to additives and neo-formed compounds, and perturbing gut microbial ecology—mechanisms not fully captured by conventional nutritional metrics (2, 9–15).

Under the NOVA framework, UPFs are not simply processed foods, but industrial formulations composed largely of refined ingredients and additives, often with little intact whole food remaining. Epidemiologic evidence now supports a robust association between higher UPF intake and cardiovascular risk. Large prospective cohorts and meta-analyses have linked greater UPF consumption with increased risks of total CVD, coronary heart disease, and stroke, although the strength of association varies across UPF subgroups. In particular, sugar-sweetened or artificially sweetened beverages and processed meats show the most consistent adverse associations, whereas other categories appear more heterogeneous. Accordingly, UPF intake should be regarded as a strong epidemiologic cardiovascular risk signal, while recognizing that most current evidence remains observational and that the mechanisms underlying this association require further causal validation.

Atherosclerosis, the dominant pathological substrate of CVD, is a chronic inflammatory and metabolic disorder characterized by endothelial dysfunction, lipid retention, immune activation, and maladaptive vascular remodeling. MicroRNAs (miRNAs) are well positioned to connect these processes because they regulate gene expression across vascular and metabolic cell types and respond dynamically to oxidative, inflammatory, glycemic, and microbial stressors (16–19). Several miRNAs central to atherosclerosis, including miR-126, miR-21, miR-33, miR-34a, miR-146a, miR-155, and miR-223, are also nutritionally responsive, supporting the concept that diet may influence vascular biology in part through miRNA remodeling (19–28).

The central hypothesis of this review is that chronic UPF exposure promotes CVD by reprogramming miRNA networks both intracellularly and across organs through extracellular vesicles (EVs). In this model, UPF-associated inflammatory, oxidative, and metabolic stress alters miRNA biogenesis and EV cargo selection, thereby amplifying intercellular communication and sustaining a feed-forward loop of endothelial dysfunction, immune activation, and plaque progression. We therefore focus not only on intracellular miRNA effects, but also on EV-mediated trafficking as a plausible long-distance signaling system linking diet-exposed organs such as the gut, liver, and adipose tissue to the vasculature.

To maintain conceptual clarity, this review is organized around the UPF–miRNA/EV–CVD axis and prioritizes synthesis over cataloging. We also distinguish mechanistic claims according to evidentiary strength—established, emerging, or speculative—to avoid overinterpretation in a rapidly evolving field.

## 2 The UPF–miRNA/EV–CVD hypothesis: a conceptual and evidence framework

The epidemiologic link between UPF intake and adverse cardiometabolic outcomes is now robust, with consistent dose–response associations reported across major cohorts and meta-analyses (5–8). However, epidemiology alone does not identify the molecular intermediates that translate dietary exposure into vascular injury. This gap is especially important because individuals with similar UPF intake may show divergent inflammatory, lipid, and vascular phenotypes, implying a gene–environment response layer not captured by standard dietary quantification.

MiRNAs offer a biologically plausible solution to this “missing link” problem. As post-transcriptional regulators, miRNAs can coordinately tune entire pathways rather than single proteins, making them ideal mediators of complex dietary exposures. In atherosclerosis, miRNAs regulate endothelial nitric oxide signaling, endothelial progenitor cell (EPC) function, vascular smooth muscle cell (VSMC) phenotype switching, macrophage polarization, cholesterol efflux, thrombosis, and plaque stability (18–20, 25, 26, 29–38). Importantly, these same miRNAs are influenced by metabolic stressors enriched in UPF-rich environments, including hyperglycemia, oxidized lipids, advanced glycation endproducts (AGEs), and inflammatory cytokines (9–13, 25–28).

A second conceptual advance is the recognition that miRNAs are not only intracellular regulators but also extracellular signals. A substantial proportion of circulating miRNAs are protected within EVs or bound to lipoproteins/protein complexes, allowing stable transport through blood and uptake by recipient cells (27, 39–41). This means that dietary injury in one tissue (e.g., gut or liver) can be “broadcast” to distant vascular beds through EV-miRNA cargo, creating a systems-level mechanism for chronic cardiometabolic propagation.

Because the current literature is heterogeneous, we apply an evidence-stratified interpretive framework throughout this review. Established mechanisms include pathways with strong experimental support across multiple models (e.g., AGE–Receptor of AGE signaling, reactive oxygen species (ROS) -associated miR-34a induction, and miR-33-mediated repression of cholesterol efflux). Emerging mechanisms include findings supported by early *in vivo* or *in vitro* data but lacking broad replication (e.g., selective EV cargo remodeling in UPF-like metabolic stress, or specific miRNAs such as miR-let-7d-5p/miR-411-5p in UPF-associated vascular phenotypes). Speculative mechanisms include plausible but insufficiently tested links between specific UPF additives (e.g., emulsifiers, nitrosamines, acrylamide) and discrete miRNA networks. This approach strengthens scientific rigor while preserving the field’s translational potential.

## 3 Core mechanistic pathways linking UPFs to miRNA remodeling and EV-mediated atherogenic signaling

The cardiovascular toxicity of UPFs appears to arise from convergence, not a single trigger. Industrial processing generates a biologically disruptive exposure pattern—high glycemic load, adverse lipid composition, additive burden, food matrix disruption, and microbiome perturbation—that repeatedly activates inflammatory and oxidative pathways known to regulate miRNA expression (2, 9–15, 42–47). This repeated signaling likely shifts the balance from vasoprotective miRNAs (e.g., miR-126, miR-143/145, miR-146a in context-dependent settings) toward pro-atherogenic miRNAs (e.g., miR-21, miR-33, miR-34a, miR-155), thereby remodeling vascular and metabolic gene programs over time.

### 3.1 Convergent inflammatory signaling as an integrated upstream pathway

**Figure 1:**
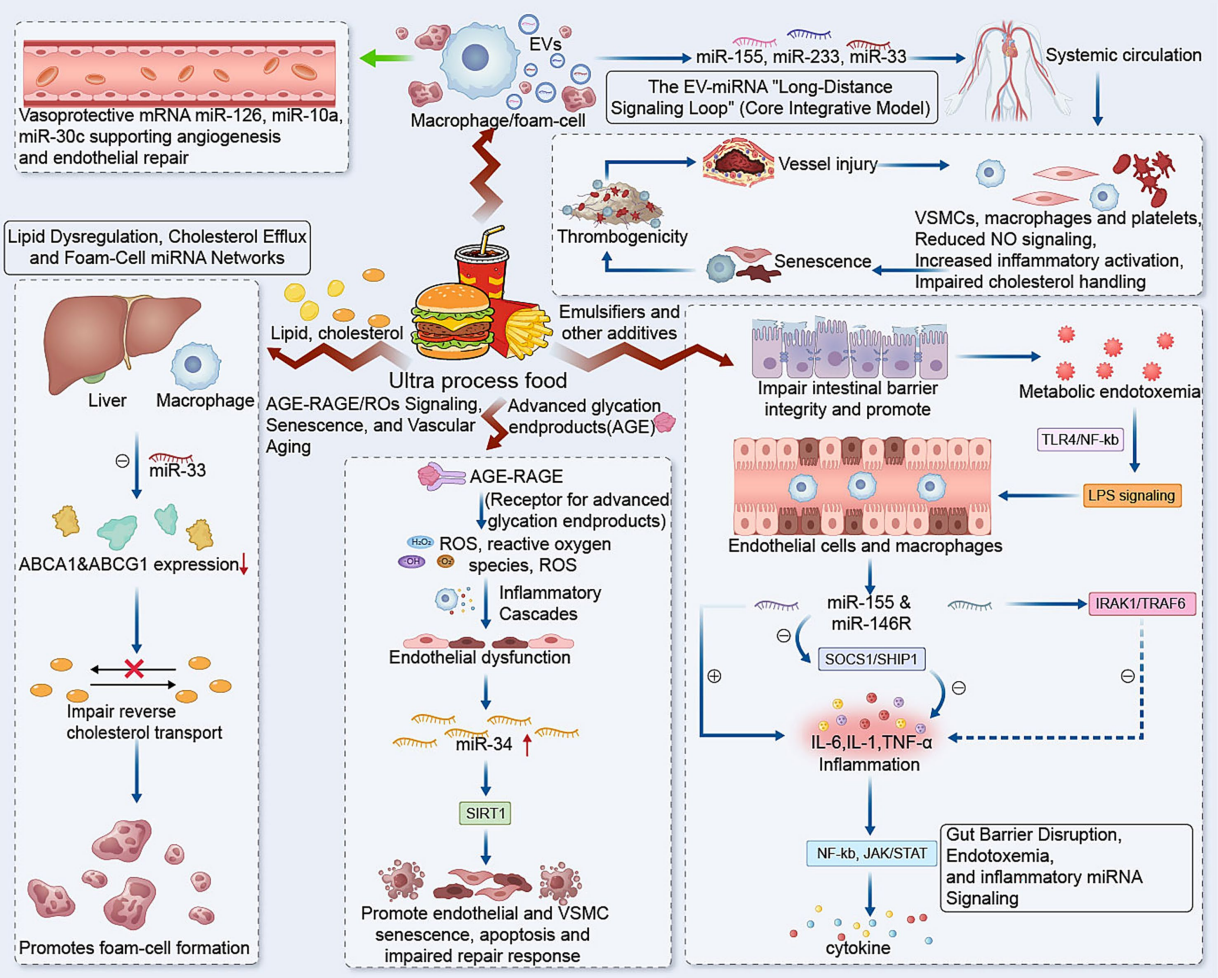
Conceptual model of how ultra-processed foods promote atherosclerotic CVD through intracellular and extracellular vesicle–associated miRNA networks. EVs, extracellular vesicles; VSMCs, vascular smooth muscle cells; AGE–RAGE, advanced glycation end products–receptor for advanced glycation end products; SOCS1, suppressor of cytokine signaling 1; SHIP1, Src homology 2 domain-containing inositol 5-phosphatase 1; IRAK1, interleukin-1 receptor-associated kinase 1; TRAF6, tumor necrosis factor receptor-associated factor 6.

A central feature of the UPF–miRNA/EV–CVD hypothesis is that multiple UPF-associated insults converge on a shared inflammatory signaling architecture rather than acting through isolated mechanisms. Emulsifier-driven gut barrier impairment, metabolic endotoxemia, microbiota disruption, and processing-related compounds such as advanced glycation end products collectively increase inflammatory tone and oxidative stress, with recurrent activation of TLR4/NF-κB- and JAK/STAT-related pathways (9–13, 45–47). This integrated upstream response provides a plausible molecular context in which nutritionally responsive miRNAs are persistently remodeled (Figure 1).

Within this framework, miR-155 and miR-146a appear especially important as counterbalancing regulators of chronic inflammatory signaling. miR-155 amplifies endothelial and macrophage activation by suppressing SOCS1/SHIP1-mediated restraint, thereby reinforcing NF-κB and JAK/STAT activity and promoting cytokine production (26). By contrast, miR-146a is generally induced as a compensatory negative-feedback signal through IRAK1/TRAF6 targeting, but prolonged low-grade inflammatory exposure may weaken or uncouple this protective brake (13, 48). The net effect is a sustained shift toward endothelial dysfunction, reduced nitric oxide bioavailability, increased adhesion molecule expression, monocyte recruitment, and macrophage activation—early events that create a permissive environment for atherogenesis.

This integrated inflammatory axis serves as the common upstream scaffold for the more specific downstream processes discussed below. Accordingly, the following sections focus on pathway-specific consequences of this shared inflammatory background, including lipid dysregulation, senescence-related vascular aging, thromboinflammation, and EV-mediated inter-organ signal propagation.

### 3.2 Lipid dysregulation, cholesterol efflux, and foam-cell miRNA networks

A parallel pathway centers on dyslipidemia and cholesterol handling. UPF-rich diets are often enriched in refined carbohydrates and unfavorable fats that can activate hepatic and macrophage lipid-sensing pathways, including those regulated by miR-33 (34, 35, 42). miR-33 suppresses ABCA1 and ABCG1, impairs reverse cholesterol transport, and promotes foam-cell formation, making it one of the best-supported mechanistic nodes linking metabolic diet stress to atherosclerosis (34, 35). Additional hepatic miRNAs, including miR-122 and miR-30c, influence triglyceride synthesis and VLDL assembly, respectively, and are likely part of the broader nutritionally responsive atherogenic network (20, 38).

Some more specific UPF-related miRNA claims remain emerging rather than established. For example, links between UPF-like hyperglycemic stress and miR-411-5p suppression, with downstream effects on endothelial adhesion and matrix remodeling, are mechanistically interesting but require broader validation. These pathways should be presented as promising hypotheses, not settled biology.

### 3.3 AGE–RAGE/ROS signaling, senescence, and vascular aging

Against this shared inflammatory background, AGE–RAGE signaling is particularly relevant because it links industrial food processing to vascular oxidative injury and cellular aging (9–11). Rather than reiterating general inflammatory activation, this pathway is most informative when considered through its senescence-related downstream effects. AGE–RAGE signaling promotes reactive oxygen species generation and induces miR-34a, which suppresses SIRT1 and thereby contributes to endothelial and vascular smooth muscle cell senescence, apoptosis, and impaired reparative capacity (25, 49). Thus, the AGE–ROS–miR-34a–SIRT1 axis represents a more specific mechanism by which UPF-related exposure may accelerate vascular aging and plaque vulnerability (Table 1).

**Table 1:** Key miRNAs implicated in UPF-related cardiovascular injury: representative targets, mechanistic roles, and level of evidence.

miRNA	Representative target(s)	Main cell type/pathway	Proposed role in UPF-related CVD	Level of evidence*	Representative refs in manuscript
miR-126	SPRED1, PIK3R2	Endothelium/EPC/EV signaling	Maintains endothelial repair, NO signaling, and vascular homeostasis; loss is linked to impaired endothelial recovery and EV-mediated dysfunction	*In vitro* + animal + human	(18, 24, 30, 40, 53, 60)
miR-92a	KLF2, KLF4	Endothelium	Promotes endothelial inflammation and reduces eNOS signaling under vascular stress	*In vitro* + animal+limited human biomarker support	(19, 23, 27, 28)
miR-10a	HMGA2	EPC/endothelium	Regulates EPC senescence and vascular repair capacity; relevance to UPF is mainly indirect/extrapolated from vascular stress biology	Primarily *in vitro*+limited animal/*ex vivo* support; no clear direct human UPF evidence	(18, 30)
miR-21	PTEN	EC/VSMC/platelet-EV axis	Promotes VSMC proliferation, endothelial stress responses, and plaque vulnerability; elevated in adverse dietary/UPF-like settings	*In vitro* + animal + human	(18, 28, 32, 53)
miR-33a/b	ABCA1, ABCG1, CPT1A	Macrophage/liver	Impairs cholesterol efflux, favors foam-cell formation, and promotes dyslipidemia in high-fat/high-UPF metabolic contexts	*In vitro* + animal + human	(27, 28, 34, 35, 53)
miR-34a	SIRT1	EC/VSMC	Links AGE/ROS stress to endothelial senescence, apoptosis, and plaque instability	*In vitro* + animal + limited human biomarker support	(25, 27, 28, 49, 53)
miR-143/145	ELK1, KLF4	VSMC	Maintains the contractile VSMC phenotype and supports plaque stability	*In vitro* + animal+limited human dietary/intervention support	(31, 40, 53)
miR-155	SOCS1, SHIP1	Macrophage/EC/EV signaling	Amplifies TLR4-NF-kB and JAK/STAT inflammatory signaling, macrophage activation, and plaque inflammation	*In vitro* + animal + human	(26–28, 53)
miR-146a	IRAK1, TRAF6, PLK2	Macrophage/EPC/inflammatory feedback	Compensatory anti-inflammatory brake on TLR/NF-kB signaling, although chronic UPF-related inflammation may uncouple this feedback	*In vitro* + animal + human	(29, 40, 48, 53)
miR-223	PSGL-1, P2Y12, CYP7A1	Platelet/liver/EV signaling	Modulates thromboinflammation, platelet-endothelial crosstalk, and bile acid/lipid handling	*In vitro* + animal + human; mechanistic direction may be context-dependent	(28, 36, 50, 51, 53)
miR-122	ADAM17, AMPKa1	Liver	Promotes hepatic lipogenesis and hypertriglyceridemia; contributes to systemic metabolic stress relevant to UPF exposure	*In vitro* + animal + human	(38, 41, 58)
miR-30c	MTP	Liver/endothelial EVs	Reduces VLDL secretion and is generally vasculoprotective; loss may favor UPF-associated dyslipidemia	Animal + limited *in vitro*/human support	(20, 24, 53)

*Level of evidence reflects the strength of support for each miRNA across the literature synthesized in the manuscript, categorized as *in vitro*, animal model, and human study. For several miRNAs, the evidence for a role in atherosclerosis/CVD biology is stronger than the evidence for a direct, UPF-specific causal mechanism; this distinction should be made clear in the table footnote or main text.

In contrast, additive-specific links to other oxidative stress-responsive miRNAs remain emerging rather than established. These mechanisms are biologically plausible, but current evidence is still insufficient to assign distinct UPF additive signatures with confidence.

### 3.4 Microbiota-related amplification, thromboinflammation, and systemic propagation

UPFs also reshape the gut microbiota, including depletion of short-chain fatty acid-producing organisms and altered host–microbial signaling (22, 45–47). In the context of the integrated inflammatory pathway described above, these microbiota-related changes are likely to function less as an independent inflammatory mechanism and more as an amplifier of systemic miRNA remodeling across immune, hepatic, and vascular compartments. This interpretation helps avoid redundancy while preserving the relevance of microbiome-mediated signaling in the overall model.

At the thromboinflammatory interface, miR-223 and miR-21 may be particularly important because they connect chronic metabolic injury to platelet activation, vascular remodeling, and plaque vulnerability (32, 36, 50, 51). Their relevance lies not in restating general cytokine signaling, but in showing how a pre-existing inflammatory-metabolic milieu may be translated into heightened event susceptibility. Direct causal mapping from specific UPF categories or additives to platelet miRNA remodeling remains incomplete and should therefore be interpreted cautiously.

## 4 Molecular mechanisms: UPF components and miRNA modulation

If intracellular miRNAs explain local pathway reprogramming, EVs explain how local injury becomes body-wide vascular pathology. EVs are lipid-bilayer particles (including exosomes, microvesicles, and apoptotic bodies) that protect RNA cargo from degradation and mediate intercellular and inter-organ communication (27, 39–41). This makes EVs highly relevant to nutritional pathobiology: they provide a mechanism by which diet-induced stress in the gut, liver, adipose tissue, or immune system can be transmitted to the vascular wall.

### 4.1 Why EVs matter in a UPF context

UPFs generate the exact stimuli known to alter EV biogenesis and cargo loading— oxidative stress, ER stress, inflammatory cytokines, glycemic surges, and lipid membrane perturbation (4, 9–11, 27, 40, 47, 52). This supports the hypothesis that chronic UPF exposure does not simply change miRNA transcription, but also changes how miRNAs are exported, protected, and delivered. In other words, UPFs may magnify the biological reach of miRNA dysregulation by increasing vesicular trafficking.

This concept is particularly important for translation. Free circulating miRNAs can be unstable and noisy, whereas EV-associated miRNAs may better reflect tissue-specific signaling states and may be more suitable for reproducible biomarker development (27, 28, 41, 53).

### 4.2 EV biogenesis and selective miRNA loading

EVs are heterogeneous. Exosomes (typically 30–150 nm) arise from multivesicular bodies; microvesicles (100–1,000 nm) bud directly from the plasma membrane; and apoptotic bodies are released from dying cells (39, 41). These subtypes differ in biogenesis, markers, and likely function, which is important because “EV-miRNA” findings are not interchangeable across isolation methods or vesicle classes.

MiRNA loading into EVs is selective rather than random, mediated by RNA-binding proteins such as AGO2, hnRNPA2B1, and YBX1 (36, 54). This selective sorting mechanism is one of the strongest conceptual supports for the EV-miRNA hypothesis: if cargo packaging is regulated, then dietary and metabolic stress could plausibly reshape the extracellular miRNA signal in a biologically meaningful way rather than merely reflecting passive cellular leakage.

### 4.3 EV sources and their likely roles in UPF-driven CVD

Endothelial-derived EVs (EEVs). Under physiologic conditions, endothelial cells release EVs enriched in vasoprotective miRNAs such as miR-126, miR-10a, and miR-30c, supporting angiogenesis and endothelial repair (24, 27). Under UPF-relevant stressors (high glucose, AGEs, inflammatory cytokines), this profile appears to shift: protective cargo declines (notably miR-126), while pro-inflammatory/pro-senescent cargo (e.g., miR-155, miR-34a, miR-92a) may increase, with downstream effects on VSMC phenotype and vascular inflammation (9, 10, 23, 24, 27, 28). This makes endothelial EVs both an early amplifier of injury and a candidate biomarker class.

Macrophage/foam-cell EVs. Activated macrophages and foam cells release EVs enriched in miR-155, miR-223, and miR-33, which can propagate inflammatory signaling, alter endothelial behavior, and impair cholesterol efflux in neighboring cells (27, 28, 36, 50, 55). These vesicles effectively convert local plaque inflammation into a mobile signal.

Hepatic and adipose EVs. Because the liver and adipose tissue are primary metabolic targets of UPF-rich diets, their EV output is likely central to systemic signaling. Hepatic EVs linked to high-fat/high-fructose states often carry lipid metabolism–related and inflammatory miRNAs (including miR-122), while adipose EVs can carry miRNAs that suppress endothelial NO signaling and increase monocyte adhesion (38, 56, 57). These vesicles provide a direct mechanism for metabolic tissue stress to shape vascular pathology.

Gut and microbiota-associated vesicles. Emerging data suggest that both host enterocytes and bacteria release vesicles that carry inflammatory signals and small RNAs. In a UPF-altered microbiome environment, bacterial outer-membrane vesicles and host-derived vesicles may transmit inflammatory signaling without requiring live bacterial translocation, offering a plausible explanation for long-range gut–vascular communication (13, 22, 47, 55).

Platelet-derived EVs. Platelets are a major source of circulating EVs. Platelet EV cargo—including miR-223 and miR-21—is well positioned to link chronic inflammation to thromboinflammation, endothelial activation, and plaque vulnerability (32, 36, 50, 58). In a UPF-exposed system already primed by metabolic and inflammatory stress, platelet EVs may help convert chronic injury into acute event risk.

### 4.4 The EV-miRNA “long-distance signaling loop” (core integrative model)

Rather than introducing a separate inflammatory mechanism, EV trafficking is best understood as the system-level propagation arm of the integrated UPF-induced signaling network. In this model, local inflammatory, oxidative, and metabolic stress is converted into circulating vesicular signals that extend vascular injury across organs and cell types.

Chronic UPF exposure induces oxidative and metabolic stress in the gut, liver, adipose tissue, and vascular compartments. These stressed tissues alter EV secretion and selectively enrich vesicles with pro-inflammatory and pro-atherogenic miRNAs (e.g., miR-155, miR-34a, miR-122, context-dependent miR-223). Once released into circulation, these EVs are taken up by endothelial cells, VSMCs, macrophages, and platelets, where they reprogram gene expression toward reduced nitric oxide signaling, increased inflammatory activation, impaired cholesterol handling, senescence, and thrombogenicity. The resulting vascular injury then drives secondary EV release from endothelial and immune cells, further amplifying systemic inflammatory signaling. Through this feed-forward loop, localized metabolic injury is transformed into sustained body-wide vascular pathology.

This “long-distance signaling loop” is especially useful because it unifies disparate observations—gut dysbiosis, hepatic lipogenesis, endothelial dysfunction, and thrombosis—into a single systems-level mechanism. It also provides a concrete rationale for both biomarker development (circulating EV-miRNA signatures) and intervention strategies (dietary remodeling, EV biogenesis modulation, or cargo-targeted therapies).

## 5 Translational relevance: human dietary signal, biomarkers, and therapeutic opportunities

### 5.1 Human intervention evidence: strong for diet–miRNA responsiveness, limited for direct UPF trials

Direct randomized human feeding studies that compare UPF-rich versus minimally processed diets while serially measuring both EV-miRNA profiles and vascular endpoints remain scarce. This is an important limitation and should be stated explicitly. However, human intervention studies already support the broader translational premise that dietary change can modulate key cardiovascular miRNAs highlighted in this review.

For example, there are human studies that provide convergent evidence for dietary modulation of miR-126, miR-155, and miR-21, even when the interventions are not framed as “UPF reduction” per se:

- miR-126: dietary intervention signals reported in blood orange juice and Mediterranean-pattern contexts (e.g., Capetini et al. (61); CORDIOPREV/Jiménez-Lucena et al. (62)), supporting endothelial miRNA responsiveness to diet quality.
- miR-155: modulation observed in polyphenol-related interventions (e.g., Daimiel et al. (63) beer intervention; Tomé-Carneiro et al. (64) resveratrol-containing grape extract), consistent with inflammatory miRNA sensitivity to dietary exposures.
- miR-21: modulation reported in phenol-enriched virgin olive oil intervention settings (e.g., Daimiel et al. (65)), consistent with effects on vascular/inflammatory remodeling pathways.

These studies should not be overinterpreted as direct evidence for UPF causality. Rather, they strengthen the translational plausibility of the central hypothesis by showing that the same miRNA nodes are modifiable in humans through nutritional interventions. This distinction is crucial: human evidence supports diet-responsiveness of the target miRNA network, while UPF-specific mechanistic causality still requires dedicated trials.

### 5.2 Biomarker translation: from circulating miRNAs to EV-miRNA panels

Circulating miRNAs, especially when measured in EV-enriched fractions, offer a plausible route to non-invasive risk stratification and adherence monitoring. Candidate panels repeatedly implicated across the current framework include miR-126, miR-155, miR-223, miR-34a, and miR-21, with additional lipid-centered markers such as miR-33 and miR-122 (27, 28, 53, 59). In principle, a composite “UPF-miRNA risk index” could complement conventional metrics (LDL-C, hsCRP, HbA1c) by capturing biologic response to dietary processing load rather than nutrient intake alone.

This approach is promising but technically demanding. Standardization of pre-analytic variables (sampling, hemolysis control), EV isolation methods, normalization strategies, and assay platforms is essential before biomarker implementation. Without such standardization, between-study differences may reflect methodology rather than biology.

### 5.3 Dietary modulation of EV biogenesis

Because miRNAs regulate coordinated gene networks rather than single targets, miRNA-directed interventions are of considerable interest in the context of UPF-related cardiometabolic injury. Preclinical studies using anti-miR-33, anti-miR-155, and miR-126 mimics have demonstrated favorable effects on lipid handling, inflammatory signaling, and endothelial repair in high-fat or hyperglycemic models (18, 24, 26, 34, 35). Although these models are not specific to UPF exposure, they engage overlapping metabolic and inflammatory stress pathways and therefore provide mechanistic support for the potential relevance of miRNA-targeted strategies in UPF-associated disease.

At the same time, dietary and lifestyle interventions are likely to represent the most scalable approaches for modulating miRNA biology in clinical practice. In this context, reducing UPF intake and adopting minimally processed or Mediterranean-style dietary patterns may be viewed not simply as general lifestyle recommendations, but as biologically active interventions capable of influencing vascular and inflammatory miRNA signatures implicated in cardiometabolic injury (51, 53, 60). Physical activity and sleep optimization may exert complementary regulatory effects on these same pathways. This perspective helps position the translational discussion within a precision nutrition framework while maintaining clear clinical relevance.

## 6 Limitations and research priorities

This field is highly promising but methodologically immature.

First, much of the current evidence is indirect. Epidemiologic studies robustly associate UPF intake with CVD, but they cannot establish miRNA-mediated causality. Mechanistic studies often use high-fat, high-fructose, AGE, or additive-focused models that only partially approximate real-world commercial UPFs. In addition, animal “UPF” diets vary widely in composition and processing intensity, limiting cross-study comparability and translational accuracy.

Second, miRNA and EV analytics remain technically heterogeneous. Pre-analytic factors (sample handling, hemolysis, RNA extraction, normalization) can materially alter circulating miRNA results, and EV isolation methods (ultracentrifugation, precipitation, size-exclusion chromatography) differ in purity and yield (16, 17, 39, 41). These issues make it difficult to compare studies and may inflate apparent biological differences.

Third, many miRNAs are pleiotropic and context-dependent. A given miRNA may be protective in one tissue or disease stage and harmful in another. This is particularly relevant for EV-miRNA interpretation, where source tissue, vesicle subtype, and recipient cell all matter. Thus, biomarker and therapeutic claims should be tissue-aware and stage-aware rather than universal.

Fourth, human intervention evidence remains stronger for “diet quality” than for “UPF-specific exposure.” The next generation of studies should therefore prioritize controlled feeding designs that directly compare UPF-rich and minimally processed diets under isocaloric conditions while measuring serial plasma/EV-miRNA profiles, vascular function, and metabolic outcomes.

Priority research directions include: (1) controlled UPF feeding trials with repeated miRNA/EV sampling; (2) EV source-tracking approaches to identify organ-of-origin signals; (3) mechanistic gain/loss-of-function experiments validating specific UPF-responsive miRNAs; (4) sex- and age-stratified analyses; and (5) multicenter standardization of EV and circulating miRNA assays. These steps are necessary to move the field from compelling association toward causal and clinically actionable science.

## 7 Conclusion

In summary, current evidence supports a plausible mechanistic link between ultra-processed food exposure and cardiovascular disease through miRNA- and extracellular vesicle–mediated pathways. Across inflammatory, metabolic, oxidative, and endothelial stress responses, miRNA remodeling may represent an important interface between dietary exposure and vascular injury. Although much of the available mechanistic evidence remains indirect and is not yet derived from UPF-specific experimental models, the overall body of epidemiologic and preclinical data supports the UPF–miRNA/EV–CVD axis as a biologically credible framework for further investigation.

These observations have important translational implications. Circulating miRNAs and EV-associated miRNAs may provide useful biomarkers of UPF-related cardiometabolic injury, while miRNA-directed interventions remain of therapeutic interest. However, the most immediate and scalable strategy remains reduction of UPF exposure together with promotion of minimally processed dietary patterns and other lifestyle factors that may modulate overlapping miRNA-regulated pathways.

Future research should focus on UPF-specific mechanistic models, longitudinal human studies with repeated biospecimen collection, standardized miRNA/EV profiling methods, and causal testing of whether dietary modification can reverse pathogenic miRNA signatures and improve cardiovascular outcomes.
